# Canadians studying medicine abroad and their journey to secure postgraduate training in Canada or the United States

**DOI:** 10.36834/cmej.68175

**Published:** 2020-07-15

**Authors:** Ilona Bartman, John R. Boulet, Sirius Qin, M. Ian Bowmer

**Affiliations:** 1 Medical Council of Canada, Ontario, Canada; 2 Educational Commission for Foreign Medical Graduates; Foundation for Advancement of International Medical Education and Research, Pennsylvania, United States; 3 (Retired) Medical Council of Canada, Ontario, Canada

## Abstract

**Background:**

From national and international workforce perspectives, Canadians studying medicine abroad (CSAs) are a growing provider group. Some were born in Canada whereas others immigrated as children. They study medicine in various countries, often attempting both American and Canadian medical licensure pathways.

**Methods:**

Using data from the Educational Commission for Foreign Medical Graduates (ECFMG) and the Medical Council of Canada (MCC), we looked at CSAs who attempted to secure residency positions in both Canada and the United States. We detailed the CSAs’ countries of birth and medical education. We tracked these individuals through their postgraduate education programs to enumerate their success rate and categorize the geographic locations of their training.

**Results:**

The majority of CSAs study medicine in one of 10 countries. The remainder are disbursed across 88 other countries. Most CSAs were born in Canada (62%). Approximately 1/3 of CSA from the 2004-2016 cohort had no record of entering a residency program in Canada or the United States (U.S.). Recently graduated CSAs were most likely to secure residency training in Ontario and New York.

**Conclusion:**

Many CSAs attempt to secure residency training in both Canada and the U.S. Quantifying success rates may be helpful for Canadians thinking about studying medicine abroad. Understanding the educational pathways of CSAs will be useful for physician labour workforce planning.

## Abstract (fr)

**Contexte:**

Selon une perspective nationale et internationale des effectifs, les Canadiens qui étudient la médecine à l’étranger (CEE) représentent un groupe en croissance. Certains sont nés au Canada, alors que d’autres ont immigré durant leur enfance. Ils étudient la médecine dans divers pays, essayant souvent parallèlement d’obtenir un permis américain et canadien pour exercer la médecine.

**Méthodes:**

À l’aide de données de l’Educational Commission for Foreign Medical Graduates (ECFMG) et du Conseil médical du Canada (CMC), nous avons examiné les CEE qui avaient tenté d’obtenir des postes de résidence à la fois au Canada et aux États-Unis. Nous avons extrait des données quant au pays de naissance et à la formation médicale de ces CEE. Nous avons suivi ces personnes dans leurs processus de demande d’admission à des programmes de formation postdoctorale pour rapporter leur taux de succès et catégoriser les emplacements géographiques de leur formation.

**Résultats:**

Nous avons identifié 10 pays d’où provenaient la plupart de ces CEE. Les autres CEE provenaient de 88 autres pays. La plupart de ces CEE sont nées au Canada (62 %). Environ 1/3 des CEE de la cohorte de 2004 à 2016 ne possède pas de dossier d’inscription à un programme de résidence au Canada ou aux États-Unis. Les CEE récemment diplômés étaient les plus susceptibles de suivre une formation en résidence en Ontario et dans l’État de New York.

**Conclusion:**

De nombreux CEE ont tenté d’obtenir un poste de résidence au Canada et aux États-Unis. Quantifier les taux de succès pourrait se révéler utile pour les Canadiens qui pensent à étudier la médecine à l’étranger. Comprendre les parcours éducatifs des CEE sera utile à la planification des effectifs médicaux.

## Introduction

Canadians studying medicine abroad (CSAs) are considered international medical graduates and must undergo the same process for selection into Canadian residency training as other international medical graduates who completed their schooling outside of Canada before becoming Canadian citizens or permanent residents (referred to in the paper as IMG[s]). The 2010 Canadian Residency Matching Services (CaRMS) study highlighted the large number of CSAs.^1^ In 2011, Watts et al. wrote that “[A] registry to monitor the number of CSAs may help provincial and federal governments, along with potential students, to plan and forecast the job market for internationally trained medical doctors” considering the “six-fold increase in the number of CSAs that was observed” between 2003 and 2010.^2^ In 2014, while underscoring the lack of statistics on the volumes of CSA, Kwong^3^ suggested that more information on CSAs, and their chances for securing residency positions in Canada, be collected and disseminated. These studies illustrate the need for information on IMGs and, specifically, on CSAs.

Although Barer et al. provided an overview and history of CSAs in Canada,^4^ and Mathews et al. looked at the realization of entry-to practice milestones by CSAs and IMG,^5^ the current manuscript expands this knowledge by providing information on the status of CSAs over time and tracking their success rates not only in Canada but also in United States (U.S.). The need to make this information available is two-fold. First, it will assist young Canadians in making informed decisions on whether to study medicine abroad and then try to return to North America for residency training. Given the comparable medical education systems and patient populations in Canada and the U.S., it is possible for the latter group to complete their residency in the U.S. and return to Canada to practise medicine. Second, since IMGs’ overall chances to secure a residency position in Canada and the U.S. are greatly impacted by the number of IMGs in the system, including CSAs, the data can help inform policies concerning the number and type of postgraduate training programs needed for internationally trained physicians.

To obtain more accurate data on CSAs, including their pathways to practice in Canada or the U.S., the Medical Council of Canada (MCC) launched a research project with the Educational Commission for Foreign Medical Graduates (ECFMG). By combining datasets from both organizations, we can describe the career pathways of CSAs, many of whom also attempt to secure residency positions in the U.S.

It is important to note the difference between the definition of a CSA as used by the MCC and ECFMG. Up to 2016, the demographic data available at the MCC allowed one to identify a CSA by country of birth only (if the country of birth was Canada, they are presumed to be a CSA). However, this form of identification omitted all Canadians born abroad but raised in Canada and holding Canadian citizenship. The ECFMG definition of a CSA includes all Canadians, regardless of their country of birth (if citizenship at entry into medical school was Canadian, they are presumed to be a CSA).

Our study cohort includes candidates who were born in Canada or were Canadian citizens at entry into medical school and entered both the Canadian and American licensure pathways that would allow them to eventually apply for a residency position in either country. In Canada, they applied for MCC Evaluating Examination (MCCEE); in the U.S., they applied for the ECFMG certification (or registered for an examination required for certification).

This study addresses the following three questions: In what countries are CSAs born and in what countries do they study? What percentage of CSAs are successful in securing residency training in either Canada or the U.S.? What are the most common training locations for these individuals?

## Study design and methods

### Data sources

The research dataset links demographic and practice information from different sources: the MCC dataset (the MCCEE and the MCC Qualifying Examination [MCCQE] Part II), the ECFMG certification dataset, and the American Medical Association (AMA) Physician Masterfile.

The MCC is the entry point for all IMGs who wish to obtain a licence to practise medicine in Canada. It administers the examinations, which are prerequisite for medical licensure in Canada. Up to 2018, all IMGs seeking a medical licence in Canada (provisional, temporary or permanent) had to pass the MCCEE. (As of 2019, MCCEE is no longer offered. Instead the MCC offers the MCCQE Part I internationally which replaced the MCCEE.) Therefore, the MCC dataset is based on this examination data. Though the first MCCEE was offered in January 1979,^6^ data became readily available in an electronic format in the early 1990s. As such, the MCCEE data used for this project begins with the 1994 administration and includes 50,024 candidates who registered for the MCCEE between 1994 and 2016.

The MCCQE Part II is a prerequisite for medical licensure in Canada. Candidates registering for this exam must have completed a minimum of 12 months of postgraduate, clinical, medical training. Consequently, the MCCQE Part II dataset includes information on postgraduate training location, which was added to the research dataset.

The ECFMG is the entry point for all IMGs in the U.S. To be eligible to enter an accredited residency program in the U.S., an IMG must be certified by ECFMG. Certification requirements include primary source verification of the medical school diploma and a passing status on the first two parts of the U.S. Medical Licensing Examination (USMLE). As part of the application, ECFMG collects data on citizenship at various stages of a candidate’s life, including at entry into medical school. Since this research focuses on CSAs, the ECFMG database was filtered for candidates who either indicated they were born in Canada or were Canadian citizens at entry into medical school. The ECFMG data includes 19,229 Canadian candidates who applied for the ECFMG certification between 1980 and 2016.

The AMA Physician Masterfile contains information on education, training and professional certification on virtually all physicians in the U.S. The dataset contains current data on approximately one million physicians and residents in the U.S. of whom approximately 25% are IMGs. For IMGs, a record is established upon entry to a postgraduate residency training program accredited by the Accreditation Council for Graduate Medical Education (ACGME).

### Participant cohort and demographic information

To compare the Canadian and American processes that CSAs undertake to secure postgraduate training, we compiled a dataset that includes candidates who, based on examination registration, were attempting to secure residency training in both countries. Thus, our final dataset included Canadian citizens who attempted the MCCEE between 1994 and 2016 and applied for the certification by the ECFMG. The MCC and ECFMG databases were linked based on name, date of birth, and gender, resulting in 7,125 matches. After the MCC-ECFMG data linkage was completed, the AMA data was added to obtain information regarding American residency training. In the final stage of the data preparation, individuals were linked to the MCCQE Part II to append information regarding Canadian residency training.

### Analyses

We conducted two sets of analyses. The first set of analyses addresses the first research question. They focused on the country of birth and on country of medical degree. For these analyses, we used the full dataset including 7,125 CSAs.

The second set of analyses addresses the second and third research questions. They focused on the postgraduate training location. We began by looking at the country of postgraduate training, Canada or U.S., and then more closely at where (e.g., province, state) in the two countries. For these analyses, we chose to focus on the most recent CSAs because our preliminary analyses of the historical CSA trend revealed the phenomenon gained momentum around 2004. To illustrate, the number of CSAs attempting the MCCEE in 2004 was 126; by 2012, the number peaked at 629. In similar fashion, in 2004, 396 ECFMG certificates were issued to CSAs; by 2013, the number peaked at 831. Consequently, the analyses of postgraduate training location included only data between 2004-2016. (N = 6,594).

Although we conducted the analyses in 2017, we chose to look at candidates who attempted the MCCEE in 2016 or earlier to allow sufficient time for individuals to apply for a residency position in Canada or the U.S. The final analysis compared the geographic region of the candidate’s medical diploma to the geographic region of their residency training.

Before presenting the results, it is important to note the postgraduate training system in the U.S. is slightly different than it is in Canada. In 1965, the Queen’s conference identified the universities’ Faculties of Medicine as the responsible parties for residency education in Canada. Therefore, residency training is funded by provincial governments but overseen by the medical schools–though some fellows and residents in programs receive external funding from international governments, research grants and/or departmental funds. Thus, when identifying postgraduate training in Canada, the location of the medical schools would be referenced. In the US, the location of postgraduate training is based on the state where the program is located and not the location of a medical school.

### Ethics approval/informed consent

Candidates registering for examinations with the MCC or ECFMG sign an agreement allowing the collected data to be used for quality assurance studies. The character of this study is quality assurance of the MCC examinee cohort, which is conducted to ensure fair and reliable examinations. As per Privacy and Confidentiality of the World Medical Association Declaration of Helsinki (2013) – Ethical Principles for Medical Research Involving Human Subjects, every precaution was taken to protect the privacy of research subjects and the confidentiality of their personal information. When the final research dataset was assembled, all identifiers were deleted; only aggregate summaries are presented.

## Results

The data in Tables 1 indicate that nearly two thirds of CSAs were born in Canada; 21.8% of CSAs were born in six other countries (India, Sri Lanka, Pakistan, Iran, United Kingdom, Poland). The remaining 15.9% were born in 108 different countries (see Table 1).

**Table 1:** Country of birth among CSAs, the frequency and percentage

Country of Birth	Frequency	Percentage
Canada	4,436	62.3
India	559	7.8
Sri Lanka	250	3.5
Pakistan	245	3.4
Iran	211	3
United Kingdom	145	2
Poland	143	2
Other	1,136	15.9
Total	7,125	100

CSAs study in many different schools around the world but three quarters of them studied in one of 10 countries; these are listed in Table 2. The remaining 22.9% studied in medical schools located in 88 countries.

**Table 2:** Most prevalent countries of medical school education among CSAs, the frequency and percentage

Country of MD Graduation	Frequency	Percentage
Ireland	1,102	15.5
Grenada	972	13.6
Saint Kitts and Nevis	786	11
Saba	722	10.1
Dominica	573	8
Poland	396	5.6
Australia	359	5
Sint Maarten	231	3.2
Netherlands Antilles	175	2.5
India	174	2.4
Other	1,635	22.9
Total	7,125	100

The second set of analyses focused on the postgraduate training location and is based on 6,594 candidates who attempted the MCCEE and applied for ECFMG certification between 2004-2016.

**Figure 1:**
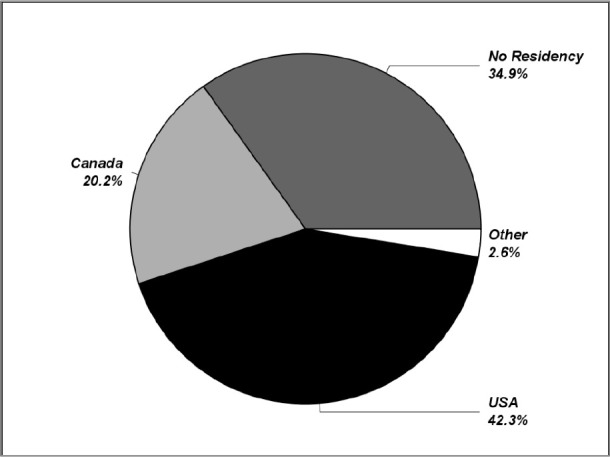
Country of residency training for CSAs, most recent 13 years (MCCEE attempt 2004 – 2016)

In the period 2004-2016, 20% of CSAs entered postgraduate training in Canada, 42% in the U.S., and 3% elsewhere. At the time of the analysis, about 35% did not manage to secure a residency position in either the U.S. or Canada (see Figure 1).

**Figure 2:**
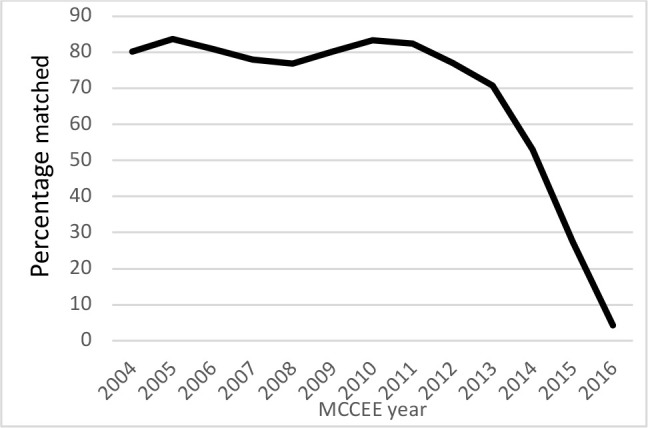
Percentage of candidates who secured Canadian or U.S. residency training program (MCCEE attempt 2004 – 2016)

Figure 2 presents the percentage of CSAs who successfully secured residency training in either Canada or the U.S. In Canada, they successfully matched to a residency training program at one of the Canadian medical schools through CaRMS. In the U.S., most residencies are secured through the National Resident Matching Program (NRMP); however, one can still be accepted to a residency position outside the NRMP Match. Figure 2 illustrates the percentage of the study cohort who secured residency training by their MCCEE attempt year.

**Figure 3:**
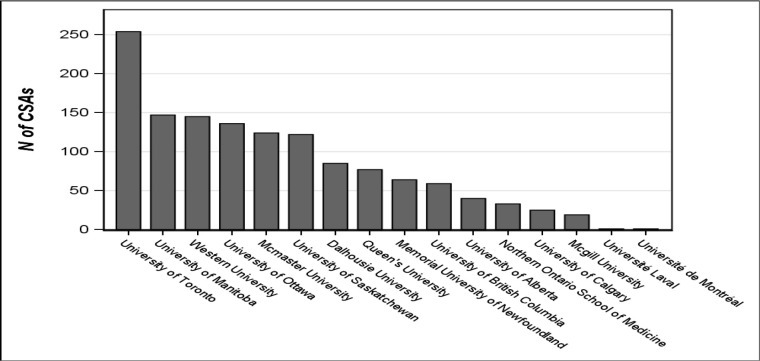
CSA placement for residency training by medical school (MCCEE attempt 2004 – 2016)

Figure 3 presents the location of postgraduate training for CSAs who secured a residency in Canada. As Canadian postgraduate medical education takes place at one of the medical schools, the CSA’s location of residency in Canada is presented by medical school.

**Figure 4:**
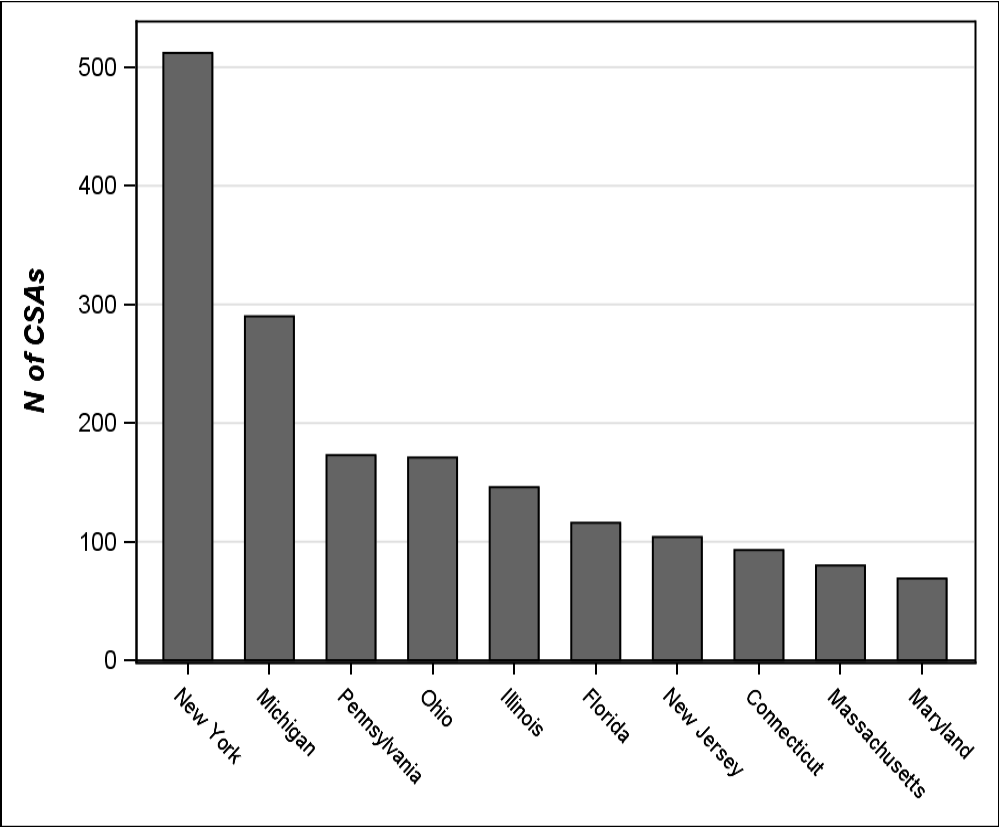
Location of postgraduate training for CSAs in the U.S. (MCCEE attempt 2004 – 2016)

Based on location of training in the U.S., New York has had the most CSA residents, followed by Michigan, Pennsylvania, and Ohio. Figure 4 presents the geographic location of CSAs who managed to secure an American residency position.

**Figure 5:**
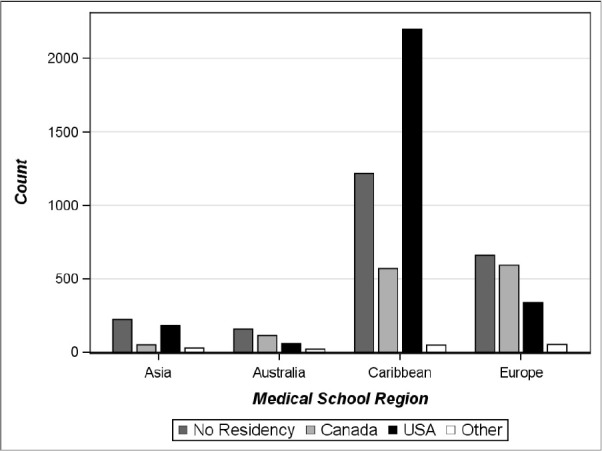
Location of postgraduate training for CSAs in Canada and the U.S. based on medical school geographic region

The final analyses compared CSAs who managed to secure a residency position in Canada to those who secured one in the U.S. and the geographic region of the medical school they attended (see Figure 5). CSAs who graduate from Caribbean schools are most likely to secure a residency position in the U.S. (N=2,198). Interestingly, those who attended a European medical school are more likely to secure a Canadian residency (N=593), although the number of European graduates and Caribbean graduates who secured a Canadian postgraduate residency position is similar (N=568).

## Discussion

Through our study we found that 38% of CSAs were born abroad. This finding is not surprising given that over 8% of Canadians under the age of 15 are foreign-born and 27% of adult Canadians are foreign-born.^7^ India, Sri Lanka, Pakistan and India have been among the top countries of origin among the Canadian immigration and they are the top countries of birth among the CSAs.

Our analyses indicate that every year hundreds of CSA apply for the MCC’s examinations and for ECFMG certification in attempt to secure postgraduate training. Looking at the 1994-2016 data, we found that CSAs study in 98 different countries; however, there are prevalent locations of medical education including Western Europe, Poland, Australia, India and the Caribbean.

Although the analyses of the location of postgraduate training revealed that 65% of our research cohort managed to secure a residency training position; the remaining one third of the cohort has no record of obtaining postgraduate training. We may, however, be underestimating the ultimate success rate since some recent candidates may be re-applying. Overall, while some CSAs may never obtain residency training, it appears that most are eventually successful. Candidates who entered the system between 2004 and 2011 have a higher success rate than those who entered the system after 2011. However, many candidates have applied several times; those who applied prior to 2012 had more opportunities to secure a position. Those who applied more recently are still queueing. We plan to re-visit the analyses in a few years to investigate whether the success rate increases if the candidates had a longer period of time to apply for a residency spot in either Canada or U.S.

Finally, we looked at the residency training location for those who secured either Canadian or U.S. residency positions. For the amalgamated data set of 2004 to 2016, 254 (19%) CSAs who managed to secure a residency spot in Canada were accepted to a residency training position at the University of Toronto. University of Manitoba and Western University each accepted 11% of the CSAs. Based on province, most CSAs train in Ontario (58%), then Manitoba (11%), and then Saskatchewan (9%). Although Ontario is home to six of the 17 medical schools in Canada and trains one third of Canadian medical students (around 950 out of 2850),^8^ the rate of acceptance per capita in Ontario is higher than one would expect. However, there may be various factors driving acceptance to residency training in other provinces such as quotas, specific speciality needs, return of service agreements, funding, etc.

In the U.S., New York residency programs have accepted the most CSAs. This is at least partially explained by the many offshore medical schools which are buying clinical clerkship positions in American hospitals. According to Helperin et al., Saint George’s University (SGU) School of Medicine and New York City’s Health and Hospitals Corporation “signed an agreement for $100 million over 10 years to purchase slots for up to 600 SGU students per year for clinical rotations.”^9^ Another example the authors provide is the American University of the Caribbean School of Medicine, which signed an agreement with New York Nassau University Medical Center for $19 million over 10 years for 64 positions. Considering there are hundreds of CSAs studying at SGU and the American University of the Caribbean, and many of these individuals will have some clinical experience in hospitals and clinics in New York, it is not surprising that New York State is the leader in CSA residency training.

As presented in this paper, CSA fill out a significant number of post-graduate training positions and consequently contribute to the physician workforce in Canada and U.S. As stated by the Health Workforce Planning Branch of the Ontario Ministry of Health and Long-Term Care, “IMGs are a valuable part of Ontario’s health care system and play an important role in providing care to Ontarians”.^10^

IMG training positions are usually return of service (ROS) positions, which means that IMGs who accept a resident position enter a contractual obligation to practice medicine in a specific area once they complete their training. Most often, ROS positions serve rural and under-serviced communities; therefore, allowing the ministries to plan around healthcare labour force distribution.^1^^1^ To illustrate, in Ontario all IMG funded resident training positions must execute a five-year ROS. Healthcare in Canada is a provincial jurisdiction; therefore, Ontario serves as an example of Canadian healthcare force planning.

However, competition among IMGs to secure a residency position in Canada is fierce. The number of dedicated IMG residency positions in Canada has been decreasing. In 2014, there were 346 dedicated positions for IMGs. In in 2015, there were 337; in 2016, there were 340^12^ (The proportion of positions available reflect only those posted on official provincial websites and do not include positions that may not have been posted or were posted only elsewhere.) At the same time the Canadian medical schools have increased their class sizes. This expansion has, however, ceased as of a few years ago (2013-2014).

Decreased number of IMG position and what may go with it decreasing number of CSA may impact the planning capacity.

## Conclusion

The purpose of this paper was to provide some insight into a specific group of IMGs in Canada, namely the CSAs. By presenting statistics on their country of birth, where they study and the success rates on securing a residency spot in Canada and the U.S., we provided additional information on the makeup of our IMG cohort. We also tried to fill out the gap on any guiding information for Canadians who are considering studying abroad. Although, many of the international medical schools tend to advertise the success rates on the variety of examinations the candidates must pass in Canada and U.S., passing these examinations does not guarantee a spot in a residency program, and a medical degree alone is not enough to practice medicine in either Canada or U.S. Considering the cost of medical education, the expanded enrolment in medical schools in Canada, and the competition among IMGs to secure a residency position, Canadians should give careful consideration when deciding whether to study medicine abroad. In our study, we found that about one third of CSAs were not successful in securing a residency position in either Canada or U.S. We are unable to comment on whether they managed to find a postgraduate training position in other countries and subsequently establishing a medical career there. However, if they plan to return to Canada at any point in the future, they would likely find it quite difficult to obtain a residency position.

Finally, we eluded to the increasing competition among IMGs, including CSA, in pursuit of post-graduate training in North America and the decreasing number of residency spots in Canada. We hope that the insight regarding the CSA cohort will be valuable to those who make plans for the Canadian Physicians Workforce

## References

[1] Canadian Resident Matching Service (CaRMS). (2010). Canadian students studying medicine abroad. Available at http://www.carms.ca/pdfs/2010_CSA_Report/CaRMS_2010_CSA_Report.pdf [Accessed on April 2016].

[2] Watts E, Davie JC, Metcalfe D (2011). The Canadian International Medical Graduate Bottleneck: A New Problem for New Doctors. Canadian Journal of Medical Education, 2(2): e8-e90. 10.36834/cmej.36566 PMC4563645 26451177

[3] Kwong W (2014). More international grads seek residency. Canadian Medical Association Journal. 10.1503/cmaj.109-4788 PMC4119154 24934892

[4] Barer ML, Evans RG, Lindsay H, (2014). Two wings and a prayer: Should Canada make it easier for Canadian doctors trained abroad to enter practice here. Healthcare Policy, 9(4): 12-19. 10.12927/hcpol.2014.23813 PMC4749883 24973480

[5] Mathews M., Kandar R., Slade S., Yi Y., Beardall S., Bourgeault I, (2017). Realization of entry-to-practice milestones by Canadians who studies medicine abroad and other international medical graduates: a retrospective cohort study. CMAJ Open. 10.9778/cmajo.20160144 PMC5498310 28630258

[6] Millar A, Dauphinee WD, (1999). The evaluating examination at twenty years, recent trends and challenges. Medical Council of Canada.

[7] Statistics Canada. Children with an immigrant background: Bridging cultures. Available at https://www12.statcan.gc.ca/census-recensement/2016/as-sa/98-200-x/2016015/98-200-x2016015-eng.cfm [Accessed in November 2019]

[8] AFMC. Canadian Medical Education Statistics (2018), Available at https://afmc.ca/sites/default/files/pdf/CMES/CMES2018-Complete_EN.pdf [Accessed in January 2020]

[9] Halperin EC, Goldberg RB (2016). Offshore medical schools are buying clinical clerkships in U.S. hospitals: The problem and potential solutions. Academic Medicine, 91(5):639-44. 10.1097/ACM.0000000000001128 26910896

[10] Ontario, Ministry of Health, Ministry of Long-Term Care. Health workforce planning branch (2019). Available at http://www.health.gov.on.ca/en/pro/programs/hhrsd/physicians/international_medical_graduates.aspx [Accessed in July 2019]

[11] Canadian Resident Matching Service (CaRMS). What is a return of service (ROS) agreement? Available at https://carms.zendesk.com/hc/en-us/articles/360002892092-What-is-a-return-of-service-ROS-agreement [Accessed in July 2019].

[12] Canadian Resident Matching Service (CaRMS). (2016). Available at https://www.carms.ca/wp-content/uploads/2018/05/table_14_dedicated_quota_offered_to_img_applicants_by_discipline_english_2016.pdf [Accessed January 2020]

